# Metagenome Analysis as a Tool to Study Bacterial Infection Associated with Acute Surgical Abdomen

**DOI:** 10.3390/jcm7100346

**Published:** 2018-10-12

**Authors:** Shao-Chun Wu, Cheng-Shyuan Rau, Hang-Tsung Liu, Pao-Jen Kuo, Peng-Chen Chien, Ting-Min Hsieh, Ching-Hua Tsai, Jung-Fang Chuang, Chun-Ying Huang, Hsiao-Yun Hsieh, Ching-Hua Hsieh

**Affiliations:** 1 Department of Anesthesiology, Kaohsiung Chang Gung Memorial Hospital and Chang Gung University College of Medicine, Kaohsiung 833, Taiwan; shaochunwu@gmail.com; 2 Division of Neurosurgery, Department of Surgery, Kaohsiung Chang Gung Memorial Hospital and Chang Gung University College of Medicine, Kaohsiung 833, Taiwan; ersh2127@cloud.cgmh.org.tw (C.-S.R.); tsai1737@cloud.cgmh.org.tw (C.-H.T.); 3 Division of Trauma Surgery, Department of Surgery, Kaohsiung Chang Gung Memorial Hospital and Chang Gung University College of Medicine, Kaohsiung 833, Taiwan; htl1688@yahoo.com.tw (H.-T.L.); hs168hs168@gmail.com (T.-M.H.); jjfce0624@gmail.com (J.-F.C.); junyinhaung@yahoo.com.tw (C.-Y.H.); 4 Division of Plastic Surgery, Department of Surgery, Kaohsiung Chang Gung Memorial Hospital and Chang Gung University College of Medicine, Kaohsiung 833, Taiwan; bow110470@gmail.com (P.-J.K.); VENU_CHIEN@hotmail.com (P.-C.C.); sylvia19870714@hotmail.com (H.-Y.H.)

**Keywords:** metagenome analysis, acute surgical abdomen, infection, ascites, blood

## Abstract

Background: The purpose of this study was to profile the bacterium in the ascites and blood of patients with acute surgical abdomen by metagenome analysis. Methods: A total of 97 patients with acute surgical abdomen were included in this study. Accompanied with the standard culture procedures, ascites and blood samples were collected for metagenome analysis to measure the relative abundance of bacteria among groups of patients and between blood and ascites. Results: Metagenomic analysis identified 107 bacterial taxa from the ascites of patients. A principal component analysis (PCA) could separate the bacteria of ascites into roughly three groups: peptic ulcer, perforated or non-perforated appendicitis, and a group which included cholecystitis, small bowel lesion, and colon perforation. Significant correlation between the bacteria of blood and ascites was found in nine bacterial taxa both in blood and ascites with more than 500 sequence reads. However, the PCA failed to separate the variation in the bacteria of blood into different groups of patients, and the bacteria of metagenomic analysis is only partly in accordance with those isolated from a conventional culture method. Conclusion: This study indicated that the metagenome analysis can provide limited information regarding the bacteria in the ascites and blood of patients with acute surgical abdomen.

## 1. Introduction

Acute abdomen is a condition that demands immediate attention and treatment. Acute abdomen may be caused by a heterogeneous group of conditions ranging from relatively benign conditions such as acute appendicitis to conditions such as diffuse peritonitis and intestinal infarction and is indicative of a surgical problem [1]. The identification of pathogenic microorganisms contaminated in the peritoneal cavity of patients with acute abdomen is important to prevent its progression into sepsis. However, the human gastrointestinal tract harbors a complex indigenous microbial flora [2,3]. Normal gut microbiota contains an estimated 10^14^ microbes [4], representing over 1000 different species of bacteria belonging to 190 different genera [5]. As the gastrointestinal tract proceeds distally, both the density and diversity of the flora increase within the colon, the majority of which reside in the host colon [6]. Although 500–600 distinct microbial species can be cultured from normal stool, conventional culture methods cultivate only 10–30% of gut microbiota [7,8]. Even in critically ill patients with sepsis, cultures were positive only in 60% of the cases [9]. In addition, the conventional culture method takes several days before the results are available and is labor intensive. Furthermore, empirical treatment with antibiotics prior to sample collection hampers bacterial growth in culture, thus compromising the sensitivity of the test.

In recent years, metagenome analysis by next-generation sequencing has emerged as a new method to identify the etiological agents of an infectious disease [10]. This method is based on the amplification and analysis of bacterial 16S rRNA genes with massively parallel processing [11]. Millions of DNA/RNA molecules in a specimen are sequenced in parallel and pathogens can be identified by matching the sequences to a reference database without bacterial cultivation [12,13]. This technology has allowed researchers to identify previously uncharacterized bacteria or viruses that cause infectious diseases [10,14,15] and also to investigate organisms previously thought to be inaccessible, including obligate anaerobes and other microorganisms that cannot survive outside their hosts without symbionts [11]. Furthermore, the metagenome analysis is not only useful for rapid bacterial diagnostics but also helps in assessing potential antimicrobial susceptibility [13]. It carries the potential to assist in using antibiotics based on an increased understanding of antibiotic-resistant genes in the gut microbiome [16,17,18].

The metagenome analysis has been performed to investigate the etiological agents of inflammatory diseases [19,20] in patients with sepsis [21], in neutropenic patients [22] and in children with central nervous system infection [23]. In a study of six patients with acute cholecystitis, the results of metagenome analysis from subjects with bacteria in bile were consistent with the results from conventional culture examination and antimicrobial susceptibility testing [13]. In a study of bacterial bloodstream infection in critically ill patients, the metagenome analysis showed significantly better diagnosis compared to the blood culture of patients who had received antibiotic pretreatment [24].

With the rapid development of applications in a clinical setting, the 16S metagenomic analysis provides an assumption-free approach to identify bacteria and has the potential to provide early diagnosis and novel treatments [16]. For example, in a clinical setting, the evaluation of cytological and biochemical components from abdominal fluid via abdominal paracentesis or diagnostic peritoneal lavage from a patient with acute abdominal disease is essential for the rapid determination of the disease etiology [25,26,27]. These results often assist the surgeon to make a decision regarding the necessity for surgery and initiate appropriate therapy [25,26,27]. Whether the additional metagenomic information from ascites could aid such decision-making merits further investigation. Using metagenome analysis, it would be interesting to answer the following questions: (1) Does the identified bacterial profile in ascites or blood reflect different etiologies of acute surgical abdomen? (2) Could bacteria in blood be indicative of those that could present in ascites? (3) Are the results of metagenome analysis in accordance with those obtained from conventional culture methods? Therefore, this study was designed to profile and compare the bacteria of ascites and blood in those patients with acute surgical abdomen using metagenome analysis.

## 2. Materials and Methods

### 2.1. Enrollment of Patients

The study protocol conforms to the ethical guidelines of the 1975 Declaration of Helsinki as reflected in an approval by the Institutional Review Board of Chang Gung Memorial Hospital (Ref: 201601132B0). One hundred patients who had acute surgical abdomen consecutively since 1 September 2016 were enrolled in this study. All patients read and signed the consent form before sample collection. Only those patients who were aged 18 years and above and those who had received laparotomy or laparoscopy were included. Exclusion criteria included patients with cancer, with an immunocompromised disease or those who had received cardiopulmonary resuscitation and those who has been intubated.

### 2.2. Clinical Data and Specimen Collection

Detailed patient information was recorded including age, sex, white blood cell count (WBC) and the percentage of segmented neutrophils which were detected at the emergency department before the operation. The operation generally started within 3 h once the diagnosis of surgical acute abdomen was established. During the laparotomy, 3 mL ascitic fluid from the abdominal cavity and 6 mL venous blood was collected from patients using EDTA tubes. The ascites were collected in the most dependent part of the abdomen or around the gall bladder. The ascites collected in the day-time were sent for DNA extraction immediately and the ascites collected in the night-time were stored at −80 °C until the next day’s morning for DNA extraction. Serum was obtained from 1 mL blood, aliquoted into 300 μL microtubes, and stored at −80 °C for cytokine analysis. All other samples were incubated with 400 μg of lysozyme (Sigma Aldrich, St. Louis, MO, USA) for 1 h at 37 °C to maximize bacterial DNA extraction. Samples of venous blood and ascitic fluid were also sent for culturing, and the isolated microorganisms were identified according to the standard microbiological procedures of the hospital [28].

### 2.3. Cytokine Analysis

The concentrations of C-reactive protein CRP (mg/L) and procalcitonin (ng/dL) in the serum were determined using the Bio-Plex^®^ system (Bio-Rad, Hercules, CA, USA) according to the manufacturer’s instructions. The serum was diluted in the ratio of 1:4 with the sample diluent and incubated at room temperature for 30 min with 300 rpm agitation to capture antibody-coupled magnetic beads. Following three washes, samples were incubated at room temperature for 30 min in the dark, centrifuged at 300 rpm and a biotinylated detection antibody, streptavidin–phycoerythrin, was added in each captured analyte and quantified using a Bio-Plex array reader.

### 2.4. Metagenome Analysis

#### 2.4.1. DNA Extraction

All DNA extractions were performed with 1 mL of ascitic fluid and blood using the QIAamp DNA Blood Mini Kit (No. 51104, Qiagen, Hilden, Germany) following the manufacturer’s protocol. The extracted DNA was eluted with recommended volume (400 μL) of elution buffer. DNA was isolated as per the manufacturer’s instructions. DNA concentration was measured by a Qubit^®^ 2.0 Fluorometer (Life Technologies, Invitrogen, Carlsbad, CA, USA).

#### 2.4.2. Amplification and Sequencing of 16S rRNA

The V3–V4 region of the bacterial 16S rRNA gene was amplified by PCR using barcoded primers reported by Klindworth et al. [29] and fused with Illumina adapter overhang nucleotide sequences. Primer sequences were 5′-TCGTCGGCAGCGTCAGATGTGTATAAGAGACAGCCTACGGGNGGCWGCAG-3′ and 5′ GTCTCGTGGGCTCGGAGATGTGTATAAGAGACAGGACTACHVGGGTATCTAATCC-3′. Two independent PCR reactions were performed for each sample. The products were pooled and indexed using Illumina’s 16S Metagenomic Sequencing Library Preparation protocol (Illumina, San Diego, CA, USA). The raw next generation sequencing (NGS) reads were first subject to quality trimming from the 3′ end, optimized for merged paired reads and fixed length trimming, followed by operational taxonomic unit (OTU) clustering before assigning a taxonomy [30]. The relative abundance based on the OTU number or taxa was used for downstream comparison of richness among groups of patients and between blood and ascites [31]. Due to possible spurious taxonomical labeling [24], those having an OTU number less than ten in both ascites and blood in all bacterial species were arbitrarily neglected in future analysis.

### 2.5. Statistical Analysis

Data were analyzed using the R statistical package version 3.3.0. The Kolmogorov–Smirnov test was used to ascertain the normality of the data. Non-normally distributed numeric variables were described by the median and interquartile range (IQR). Numeric variables were compared using the Kruskal–Wallis test to identify significantly different bacterial taxa among different groups, while categorical variables were compared by χ^2^ test. Statistical significance was indicated by two-sided *p*-values of <0.05. Hierarchical clustering of the isolates was performed using Ward’s method using Euclidean distances. The Kaiser–Meyer–Olkin (KMO) index of sampling adequacy and Bartlett’s test of sphericity were used to determine suitability of the data for dimension reduction analysis [32]. Principal components analysis (PCA) was conducted based on scree plot patterns [33] to reduce the data to a minimum number of components that could facilitate more precise data interpretation.

## 3. Results

### 3.1. Patient Characteristics

Among the enrolled patients, one patient who had a minimal amount of 16S rRNA gene in the ascites and two patients who were diagnosed as ileus were excluded from this study. Finally, a total of 97 patients with acute surgical abdomen were included and grouped into patients with peptic ulcer (*n* = 25, which included seven gastric ulcers and 18 duodenal ulcers), cholecystitis (*n* = 10), small bowel lesion (*n* = 9, which included five ischemic small bowels and four small bowel perforations), colon perforation (*n* = 4), perforated appendicitis (*n* = 30, including those with gangrene), and non-perforated appendicitis (*n* = 19, which thereafter was indicated as appendicitis) (Table 1). Those patients who had perforated or non-perforated appendicitis were significantly younger than the other groups of patients; in contrast, those patients who had peptic ulcer or colon perforation were significantly older than the other groups of patients. No significant difference was found among groups of patients regarding sex, WBC count, percentage of the segmented neutrophils and level of CRP and procalcitonin. General inflammation markers such as WBC count, levels of CRP and procalcitonin failed to distinguish specific etiology of acute surgical abdomen ranging from relatively benign conditions, such as acute appendicitis, to more sever ailments, such as colon perforation.

### 3.2. Hierarchical Clustering of the Bacteria

**Figure 1:**
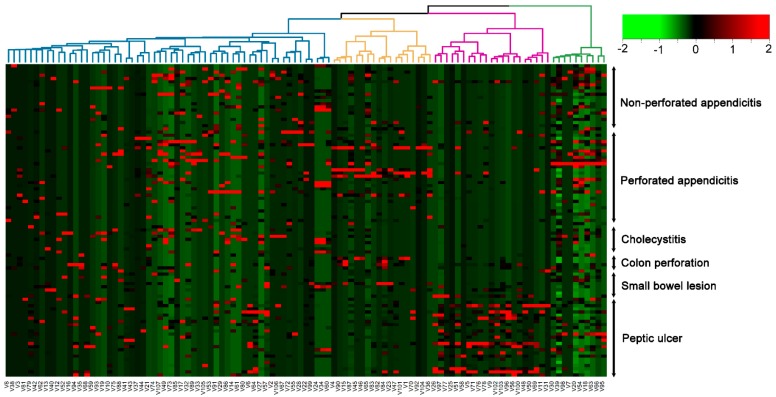
Hierarchical clustering of the 107 bacterial taxa identified from the ascites of these patients from metagenome analysis.

An overview of 107 bacterial taxa identified from the ascites of patients by metagenome analysis is shown in Supplementary Table S1. At the phylum level, bacteria were dominated by members of *Proteobacteria* and *Firmicutes*, followed by *Actinobacteria* and *Bacteriodetes*, with *Spirochaetes* and *Verrucomicrobia* in much lower numbers. Hierarchical cluster analysis (Figure 1) of bacterial communities by Ward’s method using Euclidean distances showed that patients of the peptic ulcer group had a different bacterial community.

### 3.3. Principal Component Analysis

**Figure 2:**
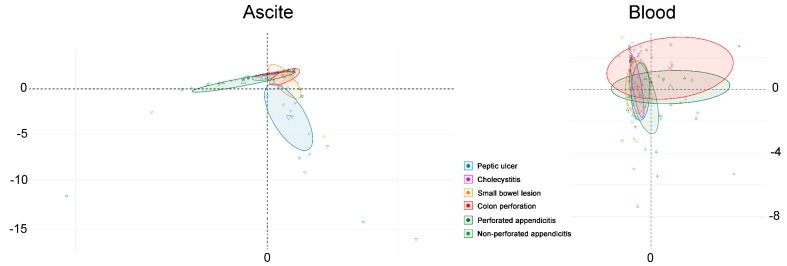
A principal component analysis conducted on the bacteria of ascites and blood to separate patients with different etiologies of acute surgical abdomen (PC1 explaining 34.1% of the variance; PC2 explaining 22.6% of the variance).

Principal component analysis (PCA) was performed on the bacteria of ascites and blood to separate patients with different etiologies of acute surgical abdomen (Figure 2). The Kaiser–Meyer–Olkin test (KMO = 0.61) and Bartlett’s test of sphericity (χ^2^ = 3642, *p* < 0.0001) [34] were used to validate that the chosen variables are able to obtain reliable and distinct factors. The resulting five-factor structure with 22 bacteria explains 77% of the total variance (Figure 1). Factor loadings of variables are shown in Figure 2. The PCA could separate the bacteria of ascites into roughly three groups: peptic ulcer, perforated or non-perforated appendicitis, and a group which included cholecystitis, small bowel lesion and colon perforation. However, the PCA could not separate the variation in bacteria of blood into different groups of patients according to their etiologies of acute surgical abdomen.

### 3.4. Correlation of Bacteria of Blood and Ascites

**Figure 3:**
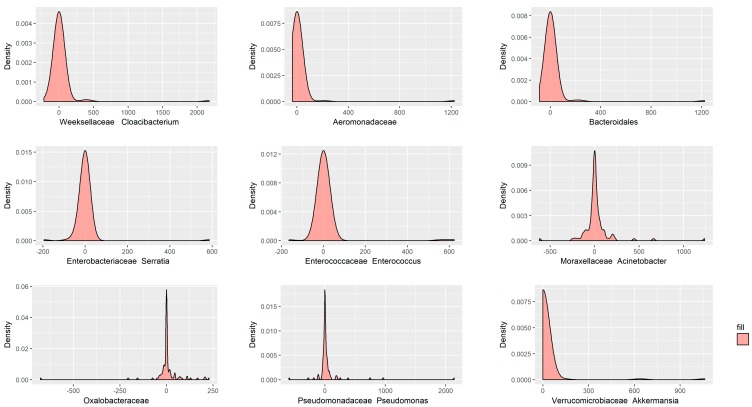
Nine bacterial taxa present a significant correlation both in the blood and ascites, with more than 500 sequence reads.

To assess whether the bacteria in the blood could indicate the bacteria that were contaminated in the ascites, the abundance of bacteria detected in blood were compared to those detected in ascites. The bacteria of blood and ascites were comparable in 48 of 107 bacterial taxa (see Supplementary Table S1) and belonged to different species. At the phylum level, the similar bacteria were dominated by members of *Proteobacteria* (16 taxa), *Firmicutes* (15 taxa), *Bacteriodetes* (11 taxa) and *Actinobacteria* (5 taxa), with *Spirochaetes* (1 taxa) in much lower frequency. Depending on the criteria of identifying at least more than 500 sequence reads in both blood and ascites, there were nine taxa showing significant correlation, including *Weeksellaceae Cloacibacterium*, *Aeromonadaceae*, *Bacteroidales*, *Enterobacteriaceae Serratia*, *Enterococcaceae Enterococcus*, *Moraxellaceae Acinetobacter*, *Oxalobacteraceae*, *Pseudomonadaceae Pseudomonas*, *Verrucomicrobiaceae Akkermansia* (Figure 3).

### 3.5. Correlation of Bacteria from Metagenome Analysis and Conventional Culture Methods

Conventional cultures of 19 ascites and three blood samples showed positive results (Table 2). These cultures of ascites and blood of patients with acute surgical abdomen showed mixed infection with Gram-positive, Gram-negative and anaerobic bacteria and did not correlate well with those identified from metagenome analysis. In the ascites, the correlation between metagenomic bacteria and conventional cultures were found as *Enterobacteriaceae* for *Escherichia coli* in nine patients or *Enterobacter cloacae* in one patient, *Klebsiella* for *Klebsiella pneumoniae* in two patients, *Neisseria subflava* for *Neisseria flavescens* in one patient, *Streptococcus* for *Streptococcus oralis* in two patients or for *Streptococcus mitis* in one patient, and *Bacteroides fragilis* for *Bacteroides fragilis* in two patients. In the blood, the correlation between metagenomic bacteria and conventional cultures could be only found as *Enterobacteriaceae* for *Escherichia coli* in one patient.

## 4. Discussion

In this study, the bacteria of ascites from metagenome analysis can be separated into three groups: peptic ulcer, perforated or non-perforated appendicitis, and a group which includes cholecystitis, small bowel lesion and colon perforation. The human appendix has been reported to harbor a robust and varied microbiota distinct from the microbiotas in other niches within the human microbiome [35]. The bacterial growth in inflamed appendices consists of a mix of aerobic and anaerobic bacteria, most often dominated by *Escherichia coli* and *Bacteroides* [36]. The metagenome analysis of microbial composition of the human appendix identified *Firmicutes* as the dominant phylum, with additional varied levels of *Proteobacteria*, *Bacteroidetes*, *Actinobacteria* and *Fusobacteria* [35]. Unsurprisingly, in those patients with peptic ulcer, the bacteria from acidic environment of the stomach may be different from those that grow in the gastrointestinal tract. It was not until the discovery of *H. pylori* in 1982 by Marshall and Warren that the acidic environment of the stomach was considered sterile [37]. However, many bacterial strains including *Streptococcus*, *Neisseria*, *Lactobacillus* and others have been repeatedly identified from the gastric fluid [38]. Most common bacteria of the stomach mucosa belong to the following five phyla: *Actinobacteria*, *Bacteroidetes*, *Firmicutes*, *Proteobacteria* (includes *H. pylori*), and *Fusobacteria* [39]. In contrast, acute cholecystitis is strongly associated with retrograde bacterial infection [13], and the bacteria are similar to those that reside in the intestine.

The results of this study revealed that the use of a bacterial profile from the ascites to reflect on different etiologies of acute surgical abdomen is still in its infancy and rather relies on PCA, which consists of varied information from specific groups of bacteria. In addition, the PCA failed to separate the variation in bacteria of blood into different groups of patients according to the different etiologies of acute surgical abdomen. This study indicates that metagenomic diagnosis may encounter some problems. First, the broad range of bacteria that can be detected limits the specificity of the assay. It is also unclear whether the detected bacteria are truly clinically significant or are environmental contaminants picked up during sample preparation. The lack of appropriate controls makes it difficult to distinguish such environmental contaminants from clinically relevant bacteria. Second, the patients are generally treated with antimicrobial agents before operation. Although the metagenome analysis is based on the sequences acquired and is regardless of dead or live bacteria, the probable impact of antibiotic use on the metagenome analysis is therefore unknown, as some information acquired from metagenome analysis may even be related to those dead bacteria and therefore be less informative. Third, the elapsed time from the occurrence of the disease to the harvesting of ascites or blood specimen for metagenome analysis may vary among some etiologies of acute surgical abdomen, especially for an illness that may have presented in an acute or subacute phase (for example, non-perforated appendicitis and cholecystitis). In such sample, the diversity of gut microbiota is significantly decreased and pathogenic bacteria would comprise the majority of gut microbiota [40]. At last, it has been reported that the intestinal microbiota from adulthood through old age changes [41], and the broad range in patient ages sustaining a varied etiology of acute surgical abdomen may lead to some bias in the analysis of the metagenomic profile. This study indicated that there still remains a space for improvement regarding the metagenomics analysis. Moreover, additional clinical manifestation or additional biochemistry biomarkers may be helpful to differentiate the various etiologies of acute surgical abdomen, such as making a difference between the cholecystitis from the perforation in the bowel.

It has been reported that 16S metagenome analysis detects more clinically significant bacteria than blood culture in children with severe febrile illness [24]. In a study of metagenomics analysis of brain abscesses, all 30 culture-positive specimens are also positive for PCR experiments [42]. In a comparative study of the bacterial pathogens from clinical specimens, it was found that metagenomics results have a concordance rate/positive predictive value of 91.8% (56/61) when compared with culture positive specimens [43]. The concordance rate would decrease to 77.3% (*n* = 75/97) when using stringent comparison criteria for metagenomics vs. culture comparison [43]. However, in this study, the results of metagenome analysis are only partly in accordance with those carried out from conventional cultures. Among the 107 bacterial taxa in the ascites, 48 taxa can be found in blood and nine taxa correlated both in the blood and ascites. We think this discordance may be partly attributed to a comparison made under relatively stringent conditions; that is, we arbitrarily neglected an OTU number less than ten in both ascites and blood in all bacterial species. Without some selection criteria, the metagenomics analysis can give a wide range of organism and microbial profiles which are difficult to interpret [43] and associated with high false-positive results [24]. Of note is the fact that the clinical features of peritonitis are dependent more on the response of the host than on the intrinsic virulence of the infecting flora [1]. For example, experimental studies suggest that the anaerobes play an important role in the induction of abscess formation, whereas the aerobic Gram-negative microbes are largely responsible for the lethality of peritonitis [44]. Some studies reported that in patients with inflammatory bowel disease, the gut microbiota show an increase in the number of species belonging to *Proteobacteria*, including *E. coli* [45], while the proportion of *Proteobacteria* in healthy human intestinal microbiota was only 1% [46]. Therefore, of concern is not only the issue of correlation of the bacterial taxa between blood and ascites, but the clinical meaning of the metagenome analysis also remains to be explored. 

So far, it is too early to say whether this technique would or would not replace conventional bacterial culture. In particular, metagenomics allows the detection of full-length antibiotic resistance genes from the Antibiotic Resistance Database [47] or from the environmental samples of unknown composition [48], making metagenomics analysis advantageous to provide timely and valuable information in prescribing antibiotics or modifying antibiotic therapy in secondary peritonitis. It is expected that, with a profound understanding of the role of the human microbiome in diseases and their interactions, as well as inter-individual differences, the metagenome analysis will progress immensely [49].

## 5. Conclusions

This study reveals that, at the current stage, metagenome analysis can only provide limited information regarding the bacteria of ascites and blood of patients with acute surgical abdomen and, although many questions still remain unanswered, there is plenty of scope for improvement of the metagenome approach to profile bacteria in surgical patients with infectious diseases.

## Figures and Tables

**Table 1:** Characteristics and inflammation profile of the patients. WBC: white blood cells.

Variables	Peptic Ulcer	Cholecystitis	Small Bowel Lesion	Colon Perforation	Perforated Appendicitis	Non-Perforated Appendicitis	*p*-Value
	(*n* = 25)	(*n* = 10)	(*n* = 9)	(*n* = 4)	(*n* = 30)	(*n* = 19)	
Female, *n* (%)	9 (36%)	6 (60%)	2 (18%)	4 (100%)	12 (40%)	10 (53%)	0.09
Age (years)	70.0 (55.0–84.0)	52.5 (45.5–57.3)	58.0 (49.0–72.0)	74.5 (63.0–80.0)	37.0 (27.5–57.8)	35.0 (31.0–42.0)	0.002
WBC (10^3^/uL)	9.4 (5.7–14.7)	12.4 (10.8–14.2)	11.6 (8.6–15.9)	10.9 (9.7–12.3)	12.4 (11.3–16.1)	13.3 (12.2–14.9)	0.20
Segment (%)	83.0 (78.0–88.4)	85.8 (82.2–87.9)	84.8 (75.6–88.0)	85.9 (78.9–91.2)	82.6 (76.5–88.2)	79.8 (76.6–84.2)	0.51
C-reactive protein (mg/L)	36.8 (2.9–99.1)	9.1 (3.9–167.3)	127.2 (47.5–240.5)	113.0 (42.2–221.3)	25.8 (18.3–55.2)	14.0 (3.8–23.0)	0.97
Procalcitonin (ng/dL)	1847.0 (11.0–3601.0)	11.3 (10.3–1895.0)	13.5 (11.0–3176.0)	12.8 (11.4–14.4)	12.0 (10.5–2743.0)	11.5 (9.5–2960.0)	0.16

**Table 2:** Comparison of metagenomic bacterium and conventional culture in the ascites and blood.

**Ascites Culture vs. Bacterium of 16S Metagenomics**			
**No.**	**Diagnosis**	**Ascites Culture**	**Bacterium**
1	Gastric ulcer	*Klebsiella pneumoniae*	*Enterobacteriaceae* *Streptococcus*
2	Duodenal ulcer	*Escherichia coli*	*Enterobacteriaceae* *Prevotella sp* *Streptococcus*
3	Duodenal ulcer	*Neisseria flavescens* *Streptococcus mitis* *Streptococcus oralis*	*Actinobacillus parahaemolyticus* *Neisseria subflava* *Streptococcus*
4	Duodenal ulcer	*Escherichia coli*	*Enterobacteriaceae* *Haemophilus parainfluenzae*
5	Duodenal ulcer	*Viridans streprococcus* *Veillonella parvula*	*Enterobacteriaceae* *Actinobacillus parahaemolyticus*
6	Duodenal ulcer	*Viridans streptococcus* *Streptococcus salicarius*	*Enterobacteriaceae*
7	Duodenal ulcer	*Klebsiella pneumoniae* *Streptococcus salivarius* *Staphylococcus aureus*	*Enterobacteriaceae* *Klebsiella*
8	Duodenal ulcer	*Streptococcus oralis*	*Enterobacteriaceae* *Streptococcus*
9	Duodenal ulcer	*Streptococcus salivarius* *Escherichia coli* *Acinetobacter*	*Enterobacteriaceae*
10	Small bowel perforation	*Escherichia coli* *Enterobacter cloacae* *Pseudomonas aeruginosa*	*Enterobacteriaceae*
11	Small bowel ischemia	*Klebsiella pneumoniae*	*Klebsiella*
12	Small bowel ischemia	*Escherichia coli* *Lactobacilus* *Bacteroides ovatus*	*Enterobacteriaceae* *Prevotella copri*
13	Colon perforation	*Escherichia coli* *Streptococcus anginosus* *Bacteroides thetaiotaomicron*	*Enterobacteriaceae* *Prevotella copri*
14	Colon perforation	*Escherichia coli* *Streptococcus asalivarius* *Klebsiella pneumoniae*	*Prevotella copri*
15	Colon perforation	*Enterococcus faecium* *Pseudomonas aeruginosa*	*Enterobacteriaceae* *Lactobacillus* *Prevotella*
16	Perforated appendicitis	*Escherichia coli* *Enterococcus avium* *Pseudomonas aeruginosa*	*Odoribacter* *Rikenellaceae*
17	Perforated appendicitis	*Escherichia coli* *Bacteroides fragilis* *Bacteroides thetaiotaomicron*	*Enterobacteriaceae* *Porphyromonas* *Bacteroides fragilis*
18	Perforated appendicitis	*Escherichia coli* *Pseudomonas aeruginosa* *Bacteroides vulgatus*	*Porphyromonas endodontalis* *Enterobacteriaceae* *Bacteroides fragilis*
19	Perforated appendicitis	*Escherichia coli* *Streptococcus anginosus* *Bacteroides fragilis*	*Enterobacteriaceae* *Porphyromonas* *Bacteroides fragilis*
**Blood Culture vs. Bacterium of 16S Metagenomics**			
**No.**	**Diagnosis**	**Blood Culture**	**Bacterium**
20	Gastric ulcer	*Roseomonas mucosa*	*Enterobacteriaceae*
2	Duodenal ulcer	*Escherichia coli*	*Enterobacteriaceae*
10	Small bowel perforation	*Staphylococcus*	*Enterobacteriaceae*

## Supplementary Materials

The supplementary material is available online at http://www.mdpi.com/2077-0383/7/10/346/s1.
